# A Retrospective Clinical Audit of Outcomes of Cochlear Implantation in Children with Multiple Disabilities in Comparison with Normal Implantees: A South Indian Experience

**DOI:** 10.1007/s12070-020-01844-7

**Published:** 2020-04-13

**Authors:** Vaishnevy Ganesh, Bhaskar Ram, Raghu Nandhan, Mohan Kameswaran

**Affiliations:** 1 School of Medicine, University of Aberdeen, Foresterhill, Aberdeen, AB25 2ZN Scotland; 2 Department of Otolaryngology, Aberdeen Royal Infirmary, Foresterhill, Aberdeen, AB25 2ZN Scotland; 3 Madras ENT Research Foundation (MERF), No.1, 1st cross street, off. 2nd main road, Raja Annamalaipuram, Chennai, Tamil Nadu 6000028 India

**Keywords:** Cochlear implantation (CI), Auditory verbal habilitation (AVH), Category of auditory performance (CAP), Speech intelligibility rating (SIR), Meaningful auditory integration scale (MAIS), Meaningful use of speech score (MUSS)

## Abstract

**Aims:**

Cochlear implantation (CI) is established as a standard remedy for children with congenital bilateral profound hearing loss to attain hearing perception and thereby develop speech and language. A subgroup includes children with multiple disabilities in whom the implant helps to improve their quality of life and also of their families via enhanced communication skills. Cochlear implants today form an integral part of their multi-handicap rehabilitation process.

**Material and Methods:**

A retrospective cohort study was carried out on children with and without multiple handicaps who have received cochlear implantation at the cochlear implant clinic of MERF, Chennai, India over the past decade. Category of Auditory Performance (CAP) scores, Speech Intelligibility Rating (SIR) scores, and also Meaningful Auditory-Integration Scale (MAIS) and Meaningful Use of Speech Scale (MUSS) scores were compared at set time frequencies of 6 months and 12 months post-implantation between the two groups of implanted children.

**Results:**

All the four CAP, SIR, MAIS and MUSS scores showed improvement over time with auditory and speech therapy in both groups of children as reflected by the improvement in their quality of life. The normative group of implantees showed better improvement compared to the group of children with multiple disabilities.

**Conclusion:**

Intensive habilitation is essential especially for children with multiple disabilities who have received cochlear implantation in which their special needs are addressed individually and optimised for the best outcome. The study shows that restoration of the special sense of hearing helps as a remedy to alleviate their other multi-handicaps to a notable extent.

## Introduction

Hearing loss in children can have devastating implications for a child’s development and speech and language acquisition thereby ending up as a double tragedy with total loss of natural communication. Children with multiple disabilities can suffer even more detrimental effects with hearing loss, wherein communication is crucial for their survival and can exacerbate other disabilities whilst putting more pressure as they would require special developmental needs and post-implantation rehabilitation [1, 2]. Treatment aimed at improving deafness can hopefully help ease other disabilities to a favourable extent.

Cochlear implantation (CI) is today recognised to play an important role in deaf children especially with multiple disabilities to help increase their quality of life and also of their families [3, 4]. In such children, CI has become an integral part in their multi-handicap rehabilitation process [5]. Madras ENT Research Foundation (MERF), Chennai, India is one of the pioneering Cochlear Implant centres in the Indian sub-continent, with more than two decades of experience in successfully rehabilitating such complex children with CI. As per Selvarajan et al. [6], this is especially since the incidence of congenital profound hearing loss in this part of the world is three times the global incidence due to the age-old practice of consanguineous marriages which has led to admixture of defective gene pools in subsequent generations.

The current project undertaken at this premier CI clinic in South India, aims to measure the outcome benefits of cochlear implantation in children with multiple disabilities by assessing their hearing-specific quality of life with auditory and speech development after cochlear implantation using standard validated outcome measures namely—Category of Auditory Performance (CAP) scores, Speech Intelligibility Rating (SIR) scores, and also Meaningful Auditory-Integration Scale (MAIS) and Meaningful Use of Speech Scale (MUSS) in children which are scales used to measure the functional benefits of cochlear implantation. The results are compared with data recorded in a comparable cohort of CI children without additional handicaps, in order to judge the level of benefit achieved in this special cohort.

## Materials and Methods

### Compliance with Ethical Standards and Conflict of Interest

Ethical approval from the Institutional Ethics Board has been obtained. There was no conflict of interest and no financial disclosures associated with this study. The authors declare that they have no conflict of interest. Inclusion of the child’s data in the study was discussed with parents and written and informed consent for participation was taken.

### Study Design and Data Source

This was a retrospective observational comparative audit of clinical outcomes that evaluated the validated habilitation scores of 40 deaf children with multiple disabilities and 40 deaf children without any additional disabilities who have received cochlear implantation over the past decade and were habilitated for one year post-implantation. The study took place at the Cochlear Implantation Clinic at MERF hospital and at the MERF Institute of Speech and Hearing (MERF-ISH) where rehabilitation sessions were given.

Data on the demographics, implantation and duration of habilitation after implantation of upto one year were gathered. Standard validated outcome measures of the pre- and sequential post-implantation CAP, SIR, MAIS and MUSS scores at set time frequencies of 6 months and 12 months were collected.

Data was obtained by reviewing patients records and from the Auditory Verbal Habilitation team’s electronic database. As per standard protocol, all implanted children attended two sessions a week with each session lasting for one hour with Auditory Verbal Habilitationists. The sequential CAP, SIR, MAIS and MUSS scores for implantees at 3 months, 6 months, 9 months and 12 months scores were documented after rehabilitation sessions as per the internationally standardised St. Gabriel’s curriculum.

### Statistical Analysis

A standardized statistical programme (SPSS v21.0, Vermont, USA) was used to evaluate the data with the help of a biostatistician associated with the CI clinic. The pre- and post-implantation scores at set-time frequencies of 6 months and 12 months were compared to normatives from a cohort of cochlear implantees without any additional disabilities to assess the degree of benefits of CI in children with multiple disabilities. A Mann-Whitley test was used to compare the two cohorts of implantees across the time scale.

### Data Availability Statement

The data that support the findings of this study are available from the corresponding author upon reasonable request.

### Results

A total of 40 deaf children with multiple disabilities and 40 deaf children from the normative cohort who have received cochlear implantation at the MERF institute were included in this study.

### Demographics of Cochlear Implantees in this Study

The mean implantation age was 3.7 years old in the group of implantees with multiple disabilities (age range = 11 months to 12 years) and 3.6 years in the control group (age range = 1–7 years). Majority of children in the multi-handicapped group were males (67.5%) (M:F = 2:1) and in the normative cohort were females (52.5%)(M:F = 0.9:1). (Table 1). 45% within the multiple disability group were diagnosed with Global Developmental Delay (N = 18) followed by Rubella (N = 8, 20%). The global developmental delay was found to be categorized into moderate to severe as per the feedback of the in-house child psychologist.

**Table 1:** Demographics of cochlear implantees in this study

Cohort of CI implantees	Children with multiple disabilities		Normative group of children	
	Mean (years)	Range (years)	Mean (years)	Range (years)
Age at onset of deafness	0	0	0	0
Duration of deafness	3.7	0.9–12	3.6	1–7
Age at implantation	3.7	0.9–12	3.6	1–7
	N (%)		N (%)	
Male	27 (67.5)		19 (47.5)	
Female	13 (32.5)		21 (52.5)	

Three out of the 40 children (7.5%) had Autism and two children suffered from epilepsy (5%). Some children suffered from Ushers syndrome (N = 1, 2.5%), Cerebral palsy (N = 2, 5%), Hydrocephalus (N = 2, 5%), Branchio-Oto-Renal (BOR) syndrome (N = 1, 2.5%), Jervell and Lange-Nielsen (JLN) syndrome (N = 1, 2.5%), Goldenhar syndrome (N = 1, 2.5%) and hypothyroidism (N = 1, 2.5%), of which some children suffered from more than one condition.

Clinical audit of outcomes:

1. **CAP score**
  75% (n = 30) of implantees with multiple disabilities scored 4 and 5 at 12 months compared to normative of 95% (n = 38) scoring above 4. A statistically significant difference is noted at 6 months (p = 0.000616) and 12 months (p = 0.000024) post-implantation between the two cohorts. (Table 2).

**Table 2:** CAP score

CAP score	Children with multiple disabilities N (%)			Normative group of children N (%)		
	Pre-implantation	6 months	12 months	Pre-implantation	6 months	12 months
0	40 (100)			40 (100)		
1		7 (17.5)	3 (7.5)			
2		11 (27.5)	5 (12.5)		6 (15.0)	1 (2.5)
3		11 (27.5)	2 (5.0)		13 (32.5)	1 (2.5)
4		11 (27.5)	18 (45.0)		14 (35.0)	8 (20.0)
5			12 (30.0)		7 (17.5)	27 (67.5)
6						3 (7.5)
7						
Total	40 (100)	40 (100)	40 (100)	40 (100)	40 (100)	40 (100)
2. **SIR score**
  17.5% and 32.5% implantees with disability scored above 3 at 6 and 12 months respectively compared to normative cohort with 25% and 55% at 6 and 12 months. A statistically significant difference noted at 6 and 12 months post-implantation intra-cohortly (multiple disabilities (p = 0.000092), normative cohort (p = 0.000001). A statistically significant difference is noted at 6 months (p = 0.004960) but not 12 months (p = 0.005243) inter-cohortly. (Table 3).

**Table 3:** SIR score

SIR score	Children with multiple disabilities N (%)			Normative group of children N (%)		
	Pre-implantation	6 months	12 months	Pre-implantation	6 months	12 months
1	40 (100)	28 (70.0)	15 (37.5)	40 (100)	13 (32.5)	2 (5.0)
2		5 (12.5)	12 (30.0)		17 (42.5)	16 (40.0)
3		6 (15.0)	5 (12.5)		8 (20.0)	10 (25.0)
4		1 (2.5)	7 (17.5)		2 (5.0)	10 (25.0)
5			1 (2.5)			2 (5.0)
Total	40 (100)	40 (100)	40 (100)	40 (100)	40 (100)	40 (100)
3. **MAIS score**
  At 6 months, 15 (37.5%) children with disabilities and 14 (35%) normative implantees scored above 21. At 12 months, 29 (72.5%) children with disabilities and 37 (92.5%) from the normative cohort achieved above 21. Statistically significant difference was noted at 6 and 12 months post-implantation intra-cohortly within the group of children with multiple disabilities (p = 8.5954E-8) and the normative cohort (p = 3.518E-8). Statistically significant difference was noted at 12 months (p = 0.030426) but not 6 months (p = 0.400656) inter-cohortly. (Table 4).

**Table 4:** MAIS score

MAIS score	Children with multiple disabilities N (%)			Normative group of children N (%)		
	Pre-implantation	6 months	12 months	Pre-implantation	6 months	12 months
0–10	38 (95)	13 (32.5)	6 (15.0)	36 (90.0)	6 (15.0)	
11–20	2 (5)	12 (30.0)	5 (12.5)	3 (7.5)	20 (50.0)	3 (7.5)
21–30		14 (35.0)	15 (37.5)	1 (2.5)	12 (30.0)	14 (35.0)
31–40		1 (2.5)	14 (35.0)		2 (5.0)	23 (57.5)
Total	40 (100)	40 (100)	40 (100)	40 (100)	40 (100)	40 (100)
4. **MUSS score**

15 (37.5%) disabled implantees and 21 (51.5%) normative implantees scored above 21 at 12 months compared to nil disabled implatees and 3 (7.5%) normative implantees at 6 months. A statistically significant difference was noted at 6 and 12 months post-implantation within the group of children with multiple disabilities (p = 2.4047E−7) and the normative cohort (p = 5.1468E−8) and at 6 months (p = 0.024955) and 12 months (p = 0.001349) inter-cohortly (Table 5).

**Table 5:** MUSS score

MUSS score	Children with multiple disabilities N (%)			Normative group of children N (%)		
	Pre-implantation	6 months	12 months	Pre-implantation	6 months	12 months
0–10	40(100)	28 (70.0)	19 (47.5)	37 (92.5)	21 (52.5)	5 (12.5)
11–20		12 (30.0)	6 (15.0)	1 (2.5)	16 (40.0)	14 (35.0)
21–30			14 (35.0)	2 (5.0)	3 (7.5)	19 (47.5)
31–40			1 (2.5)			2 (5.0)
Total	40 (100)	40 (100)	40 (100)	40 (100)	40 (100)	40 (100)

### Correlation

The intra-cohort correlation (r) value between CAP and SIR scores for the group of children with multiple disabilities is poor at 6 months (r = 0.338) and moderate at 12 months (r = 0.655), and moderate in normative cohort (6 months (r = 0.575),12 months (r = 0.690). The correlation between MAIS and MUSS is highly significant at 6 months (r multiple disabilities = 0.828, r normative = 0.781) and 12 months (r multiple disabilities = 0.812, r normative = 0.878) within each cohort.

The cross correlation between both groups of implantees is poor at 6 and 12 months (6 months: rCAP = -0.132, rSIR = 0.404, rMAIS = 0.260, rMUSS = 0.224; 12 months: r CAP = 0.049, r SIR = 0.001, rMAIS = 0.314, r MUSS = 0.164).

### Overall Speech Outcomes Acquired Post-CI with Habilitation

At the end of one year of habilitation post CI, 27 (67.5%) and 13 (32.5%) children from the group of children with multiple disabilities acquired closed-set speech and open-set speech respectively compared to the normative cohort in which and 9 (22.5%) children acquired closed-set speech 31 (77.5%) children obtained open-set speech after CI and rehabilitation.

## Discussion

This study indicated that both groups of children who received cochlear implantation showed improvement in all four outcome measures. The normative group of children displayed better scores. However, within the group of children with disabilities, there was an improvement in all the measured outcomes in keeping with the trend observed among the normal cohort although not as robust in the normative group. Thereby it is inferred that, intensive auditory verbal habilitation helps in gradually achieving optimal outcomes in the multi-handicapped cohort over time provided their special needs are well addressed in the course of their habilitation.

Cochlear implantation played an important role in rehabilitation of hearing and is especially beneficial for children with multiple disabilities to improve their auditory and speech development skills, which becomes a vital asset in the background of their other disabilities [7, 8]. Families of these implanted group of children noticed the benefits of CI as their children were more alert of their surroundings which also serves as a protective mechanism due to their better communication skills [8, 9].

Nikolopoulos et al. [10] studied the five-years post-implantation speech intelligibility in implanted children with multiple disabilities and compared it to a normative cohort of implantees. In this study, 70% of implantees with multiple disabilities achieved a SIR score of between 3 and 5 compared to 96% of the control group who achieved the same score. However, only 16% of implantees with disabilities achieved a SIR score of 4 or 5 compared to 61% of the normative cohort. This showed that multihandicapped children benefitted from CI although they did not achieve a high quality of speech, but nevertheless they are still understood in terms of speech perception [10].

The study above was in keeping with the outcome measures from this study with a global increase in all four scoring measures after 12 months of rehabilitation in both cohorts of children who received CI. The CAP, SIR, MAIS and MUSS scores in the group of children with multiple disabilities showed statistically significant difference (p < 0.05) between 6 and 12 months post-implantation with an increase in mean scores of all 4 outcomes, indicating that there is benefit with CI habilitation in this group of children but there is an apparent latent period after which the improvement recorded is tangible but also suggesting the success of the CI habilitation process.

There was a positive correlation in the outcomes measured intra-cohortly and intercohortly (except CAP score intercohortly) supporting the benefits of post-implantation rehabilitation especially in multihandicapped children. Vlahovic et al. [11] stated adequate speech perception is attainable after a prolonged course of rehabilitation in multi-handicapped children. Daneshi et al. [7] and Fukuda et al. [12] stressed that implanted children with disabilities require a specialised and continuous rehabilitation program which is in line with this study.

There is a critical age for speech and language acquisition due to neural plasticity and hearing impairment should be restored with CI prior to loss of this plasticity, especially in multi-handicapped children in whom there may be other sensory-motor losses. Hence children with hearing loss and disabilities should be recognized and managed as early as possible to reduce long term deafness effects and promote better language, intelligence and emotional development as perceived in the results of this study [9, 13–15].

Edwards et al. [16] developed the Paediatric Audiology Quality of Life questionnaire (PAQL), to assess the quality of life post-implantation for children with disabilities by parental input and 97% of children with disabilities had an improvement in quality of life post CI with relation to interaction, independence, and emotions. Hamzavi et al. [17] showed that CI children with handicaps benefitted in terms of behaviour, speech and hearing and family encouragement is essential in providing optimal benefit to children with disabilities who received implants. Other factors that can affect the outcomes of CI in children with multiple disabilities include earlier implantation age, parental counselling, intense auditory verbal therapy and rehabilitation [18]. The factors unique to the South Indian experience such as in this study are cultural differences, multi-lingual community, distance away from habilitation centres and costs for implantation and habilitation [1, 19].

The number of CI surgeries for hearing-impaired children with additional disabilities is increasing with expansion of the criteria for CI in this group of children and this should be individualised [5, 18, 20]. This complex process should involve a multidisciplinary team of experts. Understanding the patients' and family’s perception regarding the outcomes of CI is very important in determining the benefits of CI in terms of surgery and the rehabilitation process [13, 17].

In this study, a large number children with multiple disabilities were diagnosed with moderate to severe global developmental delay, but showed improvement overall with CI. Poorer speech perception post CI are associated with greater degrees of developmental delay [21]. A study by Meinzen-Derr et al. [22] showed that implanted children with developmental disabilities performed poorly in receptive and expressive language outcomes but scored highly for non-verbal cognitive abilities, highlighting the benefits of CI in this cohort of children.

Hearing-impaired children with multiple disabilities require a more intensive rehabilitation program post-CI. Restoration of the sense of hearing may help alleviate other disabilities positively and help children and their families cope better with everyday life. The difficulty in rehabilitating this cohort of children post CI has been addressed and steps should be taken to enforce CI. Special steps have been taken to address these issues within the South Indian Experience as noted in this study and as discussed in other studies done at this institution [5, 18]. These include developing a dedicated lingual map for standard rehabilitation process, having more specialised rehabilitation centres which are more accessible, more cost-effective solutions including government schemes and charitable trusts to fund the surgery, the intensive rehabilitation process, and the early diagnosis and management plans.

In summary, this study has shown improvement in both auditory and speech and language skills within the group of children with multiple disabilities compared to the normative cohort of implantees post CI. Although outcomes in these two cohorts were not similar as expected, intensive auditory verbal habilitation protocol followed at this institute has specially catered to the complex needs of the multi-handicapped children, thereby providing them satisfactory improvement with implant use over the time of habilitation and beyond. The specific issues addressed for their better outcomes have now benefitted not only these children but also their families in the long run.

## Conclusion

This study has shown that cochlear implantation in children with multiple disabilities, provides benefits in communication and listening skills. Although this is not comparable to the normative cohort of implantees, which have shown better outcomes. Nevertheless, CI can help improve the quality of life of children with disabilities and their families when coupled with proper multi-modal rehabilitation. Intensive auditory verbal habilitation is essential for children who have received cochlear implantation especially for children with multiple disabilities in which for their special needs to be addressed and optimised for the best outcome. Restoration of the special sense of hearing in this group of special children, may also be a remedy which helps to alleviate their other multi-handicaps to a notable extent, thereby offering them the window of opportunity to lead a more productive life.
